# Vanadium(IV)-Chlorodipicolinate Protects against Hepatic Steatosis by Ameliorating Lipid Peroxidation, Endoplasmic Reticulum Stress, and Inflammation

**DOI:** 10.3390/antiox11061093

**Published:** 2022-05-31

**Authors:** Yuanli Wang, Rulong Chen, Jingyi Li, Guodong Zeng, Juntao Yuan, Jingran Su, Chunyan Wu, Zhongbing Lu, Fang Zhang, Wenjun Ding

**Affiliations:** College of Life Sciences, University of Chinese Academy of Sciences, Beijing 100049, China; wangyuanli18@mails.ucas.ac.cn (Y.W.); chenrulong20@mails.ucas.ac.cn (R.C.); lijingyi20@mails.ucas.ac.cn (J.L.); zengguodong19@mails.ucas.ac.cn (G.Z.); yuanjuntao@ucas.ac.cn (J.Y.); sujingran15@mails.ucas.ac.cn (J.S.); wuchunyan18@mails.ucas.ac.cn (C.W.); luzhongbing@ucas.ac.cn (Z.L.); zhangfang@ucas.ac.cn (F.Z.)

**Keywords:** VOdipic-Cl, NAFLD, lipid metabolism, oxidative stress, endoplasmic reticulum stress, inflammation

## Abstract

Non-alcoholic fatty liver disease (NAFLD) is increasingly prevalent and represents a growing challenge in terms of prevention and treatment. The aim of this study is to investigate the protective effects and the underlying mechanisms of vanadium(IV)-chlorodipicolinate ([V^IV^O(dipic-Cl)(H_2_O)_2_, VOdipic-Cl]) in a mouse model of NAFLD induced by a high-fat diet (HFD). VOdipic-Cl (10 mg/kg/day body weight) treatment for 4 weeks significantly controlled body weight gain, and effectively reduced the increase in serum and hepatic triglyceride (TG) and total cholesterol (TC) levels, mitigated pathological injury, decreased malondialdehyde (MDA) level, and inhibited endoplasmic reticulum (ER) stress and inflammatory response in the livers of C57BL/6 obese mice. Moreover, RNA-sequencing analysis revealed distinct transcriptional profiles with differentially expressed genes (DEGs) in livers. We found that VOdipic-Cl effectively down-regulated genes related to lipid synthesis and up-regulated genes related to fatty acid transport and lipolysis, and down-regulated the expression of genes related to ER stress and immune response in the livers of obese mice. In conclusion, VOdipic-Cl effectively prevented hepatic steatosis by controlling body weight, mitigating oxidative stress, and regulating the expression of genes related to lipid metabolism, ER stress and immune response, which provides new insights into the molecular mechanism of the protective effect of VOdipic-Cl against hepatic steatosis.

## 1. Introduction

Non-alcoholic fatty liver disease (NAFLD) is the hepatic indication of metabolic syndrome [1]. During recent decades, the global prevalence of NAFLD has been estimated to be approximately 25% and has rapidly grown due to marked lifestyle changes [2]. The clinic appearance of NAFLD could be non-alcoholic fatty liver disease (NAFLD) and non-alcoholic steatohepatitis (NASH), which can increase the risk of liver fibrosis, cirrhosis, or hepatocellular carcinoma at later stages [3,4]. To date, lifestyle changes, exercise and appropriate drugs are recommended to treat this disease. Currently, emerging pharmacotherapies for the treatment of NAFLD using existing drugs mainly focus on antidiabetic, anti-obesity and antioxidants, including thiazolidinediones (e.g., pioglitazone), insulin sensitizers (e.g., metformin), or antioxidants (vitamin E) [5]. Therefore, pharmacological prevention and treatment that can effectively delay the development of NAFLD is needed.

The pathogenesis of NAFLD is complicated and multifactorial. In the liver, free fatty acids (FFAs) are consumed by β-oxidation and stored as triglycerides (TG) in lipids. The accumulation of FFAs is responsible for inducing endoplasmic reticulum (ER) stress and lipotoxicity [6,7]. On the other hand, ER stress response has been linked to obesity, diabetes and liver cancer through the induction of inflammation and insulin resistance [8]. ER stress may also be involved in the progression of NAFLD [9], as evidenced by the activation of the unfolded protein response (UPR), and was observed in the livers of obese patients with NASH [10]. Moreover, it was shown that fat accumulation sensitizes the liver to induce inflammation and cell death by oxidative stress and ER stress, culminating in NASH and fibrosis [11,12,13,14]. Those findings raised the possibility that suppression of ER stress and oxidative stress responses might benefit the treatment of NAFLD.

Vanadium compounds generally have insulin-mimetic and antidiabetic effects both in vivo and in vitro [15,16]. Our previous study has demonstrated that VOdipic-Cl alleviates hepatic lipid accumulation by inducing autophagy via the LKB1/AMPK signaling pathway in vitro and in vivo [17]. Other studies have shown that vanadate suppressed the ER stress in glial cells, primary hippocampal neurons and myocardial cells induced by tunicamycin, high sugar and Aβ [18,19,20]. Vanadium oxysulfate (VOSO_4_) reduced the low-grade systemic inflammation in type 2 diabetes mellitus (T2DM) through the reduction of both pro-inflammatory cytokines and adhesion molecules and increased adiponectin [21]. Adiponectin, as the most abundant adipose-specific adipokine, can decrease hepatic insulin resistance and attenuate liver inflammation. Generally, adiponectin predicts steatosis grade and the severity of NAFLD [22]. Therefore, vanadium compounds can regulate lipid metabolism, ER stress and inflammatory response, but the direct protective effects against NAFLD and the corresponding underlying molecular mechanisms remain largely unaddressed.

In this study, HFD-fed obese C57BL/6 mice were used as the NAFLD model. Transcriptome analyses were performed to investigate the beneficial effects of VOdipic-Cl treatment on lipid metabolism, gene expression, cell functions, and signaling pathways. qRT-PCR and Western blot were further applied to verify the RNA-seq data. Based on these studies, we trend to determine whether VOdipic-Cl could protect NAFLD as well as the underlying molecular mechanisms in case of therapeutic applications. Our data would provide significant insights for developing novel treatment strategies for NAFLD.

## 2. Materials and Methods

### 2.1. Reagents

**Figure 1:**
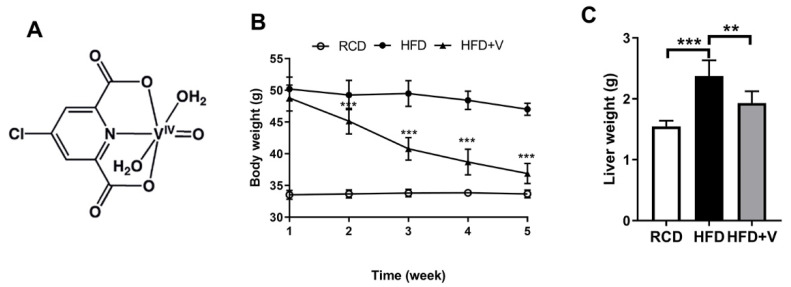
VOdipic-Cl controls the occurrence of obesity in HFD mice. (**A**) Chemical structure of VOdipic-Cl. Effects of VOdipic-Cl on body weight gain (**B**) and liver wet weight (**C**) in obese mice. Data are presented as the mean ± SD (*n* = 5). ** *p* < 0.01, *** *p* < 0.001.

VOdipic-Cl is a gift from Dr. Debbie C. Crans (Colorado State University, Fort Collins, Colorado, USA). The schematic structure of the VOdipic-Cl compound used in this study is shown in Figure 1A. TG, TC, FFAs, low density lipoprotein cholesterol (LDL-c), high density lipoprotein cholesterol (HDL-c), alanine aminotransferase (ALT) and aspartate aminotransferase (AST), MDA, glutathione/oxidized glutathione (GSH/GSSG), 3′-nitrotyrosine (3′-NT) assay kits were purchased from Nanjing Jiancheng Bioengineering Institution (#A110-1-1, #A111-1-1, #A042-2, #A113-1-1, #A112-1-1, #C009-2-1, #C010-2-1, #A003-1-100, #A061-2-1, #MU30862, Beijing, China). A superoxide dismutase (SOD) assay kit was purchased from Beijing Solarbio Science & Technology Co., Ltd. (#BC0175, Beijing, China). Dihydroethidium (DHE) was purchased from Shanghai Yisheng Bio-Technology Co., Ltd (#RM2235, Beijing, China). TRIzol reagent was purchased from ThermoFisher (#15596-026, Waltham, MA, USA).

### 2.2. Animal Experiments

Male C57BL/6 mice (*n* = 15, 22–24 g, obtained from Huafukang Biotechnology Co., Beijing, China) were maintained under a controlled temperature (22 ± 2 °C) and relative humidity (40–60%) with a 12 h light/dark cycle, and fed commercial regular chow diet and distilled water ad libitum. The study protocol was approved by the Animal Ethics Committee of the University of Chinese Academy of Sciences (Beijing, China). The research was conducted in accordance with the Guide for the Care and Use of Laboratory Animals as adopted and promulgated by the United States National Institutes of Health.

Various overnutrition-based models have attempted to mimic the hepatic histological features similar to human NAFLD pathogenesis. Among them, a high-fat diet is used to build NAFLD animal models [23]. After 1 week of acclimatization, animals were randomly divided into three groups of five mice per group. These were regular chow diet (RCD), high-fat diet (HFD), and VOdipic-Cl treatment (HFD+V) groups. The RCD group was fed with a standard laboratory chow (D12450B, Huafukang Biotech Co., Beijing, China). The other groups were fed with HFD containing 60% fat, 20% protein, and 20% carbohydrate (H10060, Huafukang Biotech Co., Beijing, China). All groups had free access to water and food. After 20 weeks of feeding, the RCD and HFD animals were given oral gavage daily with distilled water. The HFD+V group animals were daily administered with VOdipic-Cl (10 mg/kg body weight) for 28 days. All animals were sacrificed, with blood and liver samples collected for further analysis. The blood samples were centrifuged at 500× *g* for 15 min at 4 °C to obtain the serum. The liver of each mouse was immediately excised, weighed and stored in liquid nitrogen until analysis.

### 2.3. Biochemical Parameter Tests

Biochemical parameters, including the concentrations of TG, FFAs, TC, LDL-c, HDL-c, ALT and AST in serum, were determined using commercially available enzyme-linked immunosorbent assay (ELISA) kits according to the manufacturer’s instructions. Fresh liver tissues (50 mg of each sample) were extracted and homogenized using a tenfold volume of ethanol. Then, 10 μL of supernatant was taken for determination after centrifugation. The contents of TG, TC, 3′-NT, MDA, SOD, and GSH/GSSG in hepatic tissues were evaluated following the manufacturer’s instructions.

### 2.4. Histopathological Examination

In brief, liver tissue samples (0.2 cm × 0.2 cm) were fixed with 4% paraformaldehyde for 24 h and then processed for paraffin embedding. Embedded tissue blocks were serially sectioned at 5 μm thickness using a rotary microtome (Leica RM 2135, Wetzlar, Germany) and stained with hematoxylin and eosin (H&E) to assess hepatic steatosis.

In addition, liver tissue samples were also embedded with SAKURA Tissue-Tek^®^ O.C.T. compound, and then quickly frozen in liquid nitrogen. The embedded tissue blocks were sectioned at 7 μm thickness using a frozen slicer (Leica CM3050S, Wetzlar, Germany) and stained with oil red O or dihydroethidium (DHE) for 30 min to assess lipid accumulation and superoxide generation, respectively. Five mice per group were used for these experiments.

The results of H&E and oil red O staining were photographed and recorded with an upright fluorescence microscope (Leica DM6000 B, Germany) and analyzed by Image J software. The DHE fluorescence staining was examined by confocal microscopy (LSM880, Zeiss, Germany) at excitation and emission wavelengths of 561 and 640 nm, and ana-lyzed with Zen 2 software (Carl Zeiss, blue edition).

### 2.5. RNA-Sequencing (RNA-seq)

Total RNA was extracted from the livers of RCD, HFD or HFD+V groups using TRIzol reagent (3 mice per group). RNA quality was determined by Agilent 2100 bioanalyzer (ThermoFisher Scientific, Waltham, MA, USA), and samples with an RNA integrity number above 8 were used for subsequent experiments. Library construction and RNA sequencing were performed on a BGISEQ500 platform (BGI-Shenzhen, China). The sequencing data for clean reads generated by this study have been deposited in the NCBI Sequence Read Archive database (accession number: PRJNA540011). Then the clean reads were mapped to the reference genome (Mus_musculus, GCF_000001635.25_GRCm38.p5) using Hierarchical Indexing for Spliced Alignment of Transcripts (HISAT) [24] or Bowtie 2 software [25]. The matched reads were calculated and then normalized to reads per kilobase per million mapped reads (RPKM) value using RNA-Seq by Expectation Maximization (RESM) software to obtain the gene expression level [26]. The differential expression of genes (DEGs) between two groups was screened by DEGseq [26] with the following thresholds of fold change ≥2 and adjusted Q value ≤ 0.05.

To further understand the biological functions of genes, the identified DEGs in each pair were mapped to terms in the Kyoto Encyclopedia of Genes and Genomes (KEGG) database (http://www.genome.jp/kegg/pathway.html, accessed on 16 May 2022). In addition, we performed enrichment analysis using the cluster Profiler and ggplot2 function of R software. Gene set enrichment analysis (GSEA) was also performed using the KOBAS 3.0 online tool 1 and Java GSEA2. The P-value was adjusted for false discovery rate (FDR) to get Q-value, and Q-value ≤ 0.05 was considered significant enrichment.

### 2.6. Quantitative Real-Time PCR and Western Blot Analyses

The cDNA was synthesized using PrimeScript™ RT Master Mix (RR036B, TaKaRa, Otsu, Japan). A quantitative real-time polymerase chain reaction (qPCR) was performed using TB Green^®^ Premix Ex Taq™ II (#RR820DS, TaKaRa). Primer sequences for validation of genes by qPCR are shown in Table S1.

Biyuntian RIPA cell lysis solution (# P0013B, Shanghai, China) was used for ice lysis of cells for 30 min, centrifuged at 12,000× *g* at 4 °C for 20 min, and the supernatant was collected for Western blot analysis. Detailed information on antibodies is shown in Table S2.

### 2.7. Statistical Analysis

The results were expressed as the mean ± standard deviation (SD). The significance of group differences was analyzed using GraphPad Prism 8 (GraphPad Software Inc., CA, USA) with the independent t-test. Multiple comparisons were performed using one-way ANOVA following the post-hoc Tukey test. Statistical significance was defined as *p* < 0.05.

## 3. Results

### 3.1. VOdipic-Cl Regulates Body Weight and Liver Weight in Obese Mice

During model establishment, the bodyweight of mice in each group continued to increase. After 20 weeks, mice fed with HFD were significantly heavier than the control (Figure 1B). After 4 weeks of VOdipic-Cl treatment, the body weight and liver wet weight of mice in the HFD+V group were significantly less than those in the HFD group (Figure 1C). The results indicated that VOdipic-Cl controlled the occurrence of obesity in HFD mice.

### 3.2. VOdipic-Cl Ameliorates HFD-Induced Liver Steatosis in Obese Mice

**Figure 2:**
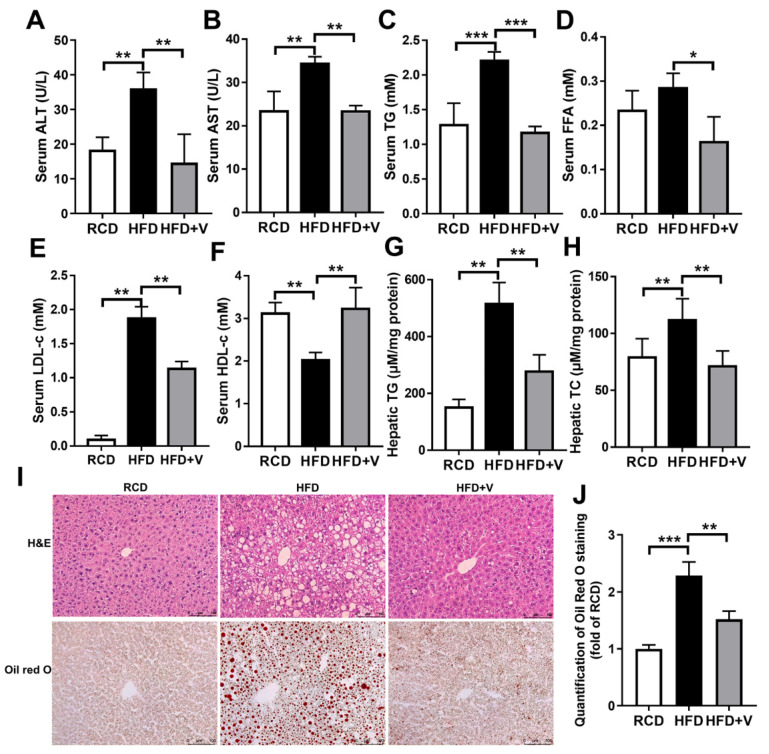
Hepatoprotection of VOdipic-Cl against HFD-induced liver steatosis. Serum ALT (**A**), AST (**B**), TG (**C**), FFA (**D**), LDL-c (**E**), HDL-c (**F**), as well as hepatic TG (**G**) and TC (**H**) levels elevated by HFD were significantly decreased by treatment with VOdipic-Cl. (**I**) Representative H&E and oil red O stained sections of hepatic tissues (scale bar = 100 μm). (**J**) Statistical analysis results of oil red O staining. Data are presented as mean ± SD (*n* = 5). *p < 0.05, ** *p* < 0.01, *** *p* < 0.001.

The levels of liver transaminases and blood lipid are key indicators of NAFLD. As shown in Figure 2A–H, the HFD caused severe liver function damage as evidenced by increases in serum ALT and AST (Figure 2A,B), TG, FFA, LDL-c (Figure 2C–E) and a decrease in serum HDL-c (Figure 2F). Moreover, hepatic TG and TC were significantly increased by HFD (Figure 2G,H) compared to the control mice. However, VOdipic-Cl treatment significantly reduced the increase of liver transaminase levels and serum and hepatic lipid caused by HFD. Furthermore, histological examination of H&E and oil red O stained sections of hepatic tissues showed large lipid droplets, balloon-like degeneration, partial necrosis, multiple inflammatory aggregations, and severe steatosis of hepatocytes. The analysis of TG and TC levels in the hepatic tissue of mice in the HFD group supported the histopathology results. However, VOdipic-Cl treatment significantly reduced the signs of liver injury and decreased the extent of adipose infiltration (Figure 2I,J). These findings showed that VOdipic-Cl inhibited hepatic fat synthesis and ameliorated liver function damage.

### 3.3. VOdipic-Cl Attenuates HFD-Induced Hepatic Oxidative Stress

**Figure 3:**
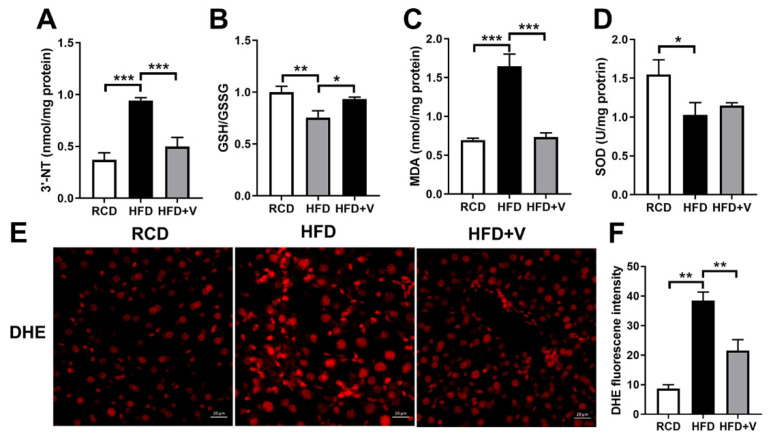
VOdipic-Cl attenuates hepatic oxidative stress in the livers of obese mice. (**A**–**C**) 3′-NT, MDA and GSH/GSSG levels. (**D**) SOD activity. (**E**,**F**) Liver sections were stained with DHE, and the relative DHE fluorescence intensities were determined. Scale bar = 20 μm. Data are presented as mean ± SD (*n* = 3). * *p* < 0.05, ** *p* < 0.01, *** *p* < 0.001.

It has been proposed that fatty acid accumulation in hepatocytes is induced by high concentrations of fatty acids due to lipolysis and the associated oxidative damage in the liver. [27] To evaluate the protective effect of VOdipic-Cl on hepatic oxidative stress in obese mice, we firstly determined GSH/GSSG level and SOD activity and the marker of lipid peroxidation, including MDA, 3′-NT, and DHE. As shown in Figure 3A–D, an increase of 3′-NT, MDA, and a decrease of GSH/GSSG and SOD in the HFD group were observed compared to the RCD group. However, the extent of changes was ameliorated by VOdipic-Cl (Figure 3A–D). In addition, DHE staining revealed that VOdipic-Cl decreased superoxide levels in the livers of HFD mice (Figure 3E,F), indicating that VOdipic-Cl treatment alleviated HFD-induced lipid peroxidation and increased antioxidant capacity in the livers of NAFLD models.

### 3.4. VOdipic-Cl Affects Gene Expression Profile in the Livers of Obese Mice

**Figure 4:**
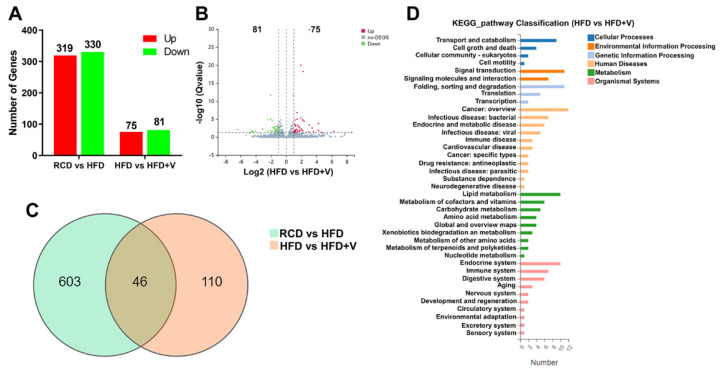
Analysis of differentially expressed genes between RCD and HFD-fed mice livers. (**A**) Summary of the number of up- and down-regulated genes in the RCD vs. HFD group, and HFD vs. HFD+V group. Fold change ≥ 2 and adjusted Q value ≤ 0.05 were used as the threshold to judge the significance of gene expression difference. (**B**) The fold changes of DEGs in the HFD vs. HFD+V group were visualized by volcano plot. (**C**) The number of different and overlapped DEGs from the RCD vs. HFD group, and the HFD vs. HFD+V group are illustrated in the Venn diagram. (**D**) KEGG classification of DEGs from HFD vs. HFD+V groups. The functions of genes identified cover six main categories: cellular processes, environmental information processing, genetic information processing, human disease, metabolism and organismal system (*n* = 3).

To further characterize the mechanisms underlying the alleviating effects of VOdipic-Cl on hepatic steatosis, RNA-seq was performed to analyze and compare the transcriptome differences in the hepatic tissues between RCD, HFD, and HFD+V groups (Figure 4). A total of 649 DEGs (319 up-regulated and 330 down-regulated) were identified when comparing the RCD group and HFD group, and the fold change of these DEGs was visualized by a volcano plot (Figure 4A and Figure S1A). The top 10 up- and down-regulated genes are listed in Table 1. Among them, *Cidea*, *Sptlc3*, *Mogat1*, *Acsm2*, *Cyp2b9*, *Cyp2c40,* and *Cyp26A1* mainly enriched the regulation of lipid metabolism and lipid oxidation. We also identified 156 DEGs (75 up-regulated and 81 down-regulated) from HFD vs. HFD+V group (Figure 4A), and the fold changes of these DEGs were visualized by a volcano plot (Figure 4B). The top 10 up- and down-regulated genes are listed in Table 2. Among these genes, *Gsta1* and *Gm3776* are related to oxidative stress. *Ctsg*, *Elane*, *Cxcl13* and *Bcl6* are related to inflammation.

Next, we created a Venn diagram to visually depict the similarities and differences between the DEGs in each pair. As shown in Figure 4C, there were 46 overlapped DEGs between RCD vs. HFD+V and HFD vs. HFD+V groups. According to the KEGG annotation and official classification, we mapped the DEGs of each pair to KEGG pathways, and the results were presented in Figure S1B and Figure 4D. Among these perturbed pathways, signal transduction, lipid metabolism, endocrine system, and immune system have the highest DEGs. We then performed KEGG pathway enrichment analysis. We found that many DEGs from the RCD vs. HFD groups were mainly enriched in metabolism pathways, genetic information processing, and immune pathways, such as steroid biosynthesis, fatty acid metabolism, peroxisome proliferator-activated receptor (PPAR) signaling pathways, and protein processing in endoplasmic reticulum pathways (Table 3 and Figure S2A). We also found that the DEGs of the HFD vs. HFD+V group were significantly enriched in protein processing in the endoplasmic reticulum, steroid biosynthesis, retinol metabolism, and chemical carcinogenesis pathways (Table 4 and Figure S2B).

### 3.5. Effect of VOdipic-Cl on the Expression of Hepatic Triglyceride Synthesis and Lipolysis Genes in Obese Mice

**Figure 5:**
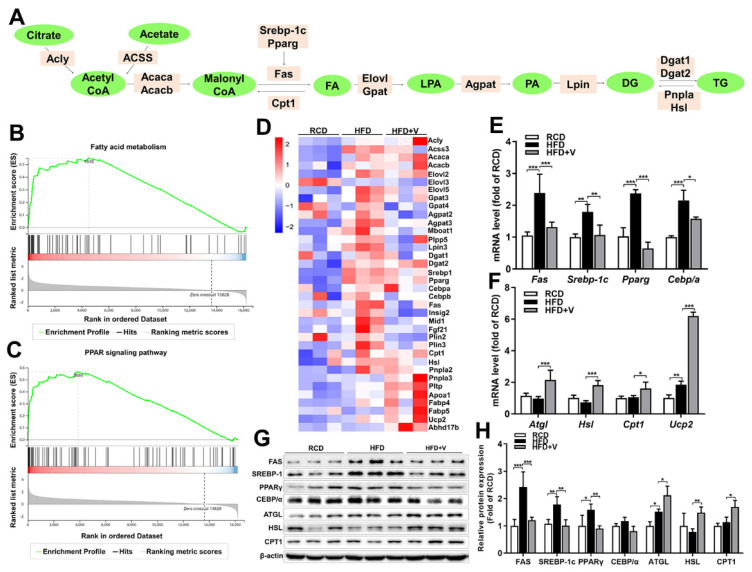
Effect of VOdipic-Cl on the expression of hepatic triglyceride synthesis and lipolysis genes in obese mice. (**A**) Schematic of the hepatic de novo lipogenesis pathway, where genes are presented in pink and metabolites in green. (**B**,**C**) GSEA-KEGG gene set enrichment analysis plot showing the DEGs in the RCD vs. HFD group mainly enriched in fatty acid metabolism and PPAR signaling pathway. (**D**) The gene expression profiles of the DEGs that were mainly enriched in lipid metabolic pathways, as determined by KEGG pathway analysis, are shown in the heat map. (**E**,**F**) The mRNA levels of lipid synthesis- and lipolysis-related genes. (**G**,**H**) The protein levels of genes are related to lipid metabolism. Data are presented as mean ± SD (*n* = 3). * *p* < 0.05, ** *p* < 0.01, *** *p* < 0.001.

Having determined that VOdipic-Cl effectively reduces hepatic TGs in obese mice, we next sought to establish whether genes involved in TG or fatty acid production were regulated in response to VOdipic-Cl treatment. Hepatic de novo lipogenesis includes the coordinated actions of several enzymes that synthesize fatty acids from two-carbon precursors [28] and then conjugate these fatty acids to a glycerol backbone to yield TGs containing various acyl chains [29]. The enzymes and metabolites of this pathway are exhibited in Figure 5A. To better understand the role of VOdipic-Cl in controlling elevated hepatic lipid levels, RNA-seq was performed to analyze the hepatic mRNA levels of genes encoding all the enzymes in RCD, HFD and HFD+V groups. The analytical results of the GSEA-KEGG pathway analysis showed that HFD significantly up-regulated the expression of genes related to the pathway of fatty acid metabolism and the PPAR signaling pathway (Figure 5B,C). The expression profile of the DEGs in metabolic pathways is shown in the heat map (Figure 5D). Compared with the RCD group, the expression profile of some lipid synthesis related genes, including acetyl-coenzyme a synthetase 2-like *(Acss),* acetyl-coa carboxylase 1 *(Acaca),* elongation of very-long-chain fatty acids protein 5 *(Elovl5),* glycerol-3-phosphate acyltransferase *(Gpat),* diacylglycerol acyltransferase 1 *(Dgat1),* fatty acid synthase *(Fas),* sterol regulatory element-binding protein 1c *(Srebp1c),* peroxisome proliferator-activated receptor γ *(Pparγ),* CCAAT enhancer-binding proteins α *(C/EBPα),* insulin-induced gene 2 *(Insig2),* mindline1 *(Mid1),* were up-regulated in the HFD group. However, these genes were significantly down-regulated after VOdipic-Cl treatment. In addition, the expression of genes related to fatty acid transport and lipolysis, including apolipoprotein a1 (*Apoa1),* fatty acid-binding protein 4 *(Fabp4), Fabp5,* patatin like phospholipase domain containing 2 *(Pnpla2/Atgl),* hormone-sensitive lipase *(Hsl)* and uncoupling protein 2 (*Ucp2)* were also up-regulated by VOdipic-Cl. Furthermore, we performed qPCR to validate the changes of some genes, including *Fas*, *Srebp1c*, *Pparγ*, *Cebp/α, Atgl, Hsl,* carnitine palmityl transferase 1 *(Cpt1), and Ucp2.* We found that VOdipic-Cl significantly inhibited the up-regulation of *Fas*, *Srebp1c*, *Pparγ*, and *Cebp/α* induced by HFD (Figure 5E) and increased the expression of *Atgl, Hsl, Cpt1 and Ucp2* (Figure 5F). Therefore, the results demonstrated that VOdipic-Cl treatment significantly down-regulated the expression of genes related to lipid synthesis and up-regulated the expression of genes related to lipolysis and fatty acid oxidation. Western blotting results further showed that VOdipic-Cl treatment significantly reduced the protein expression levels of FAS, SREBP-1C, PPARγ, and CEBP/α, and increased the protein expression levels of ATGL, HSL, and CPT1 (Figure 5G, H).

### 3.6. Effect of VOdipic-Cl on the Gene Expression Profile of Hepatic ER Stress in Obese Mice

**Figure 6:**
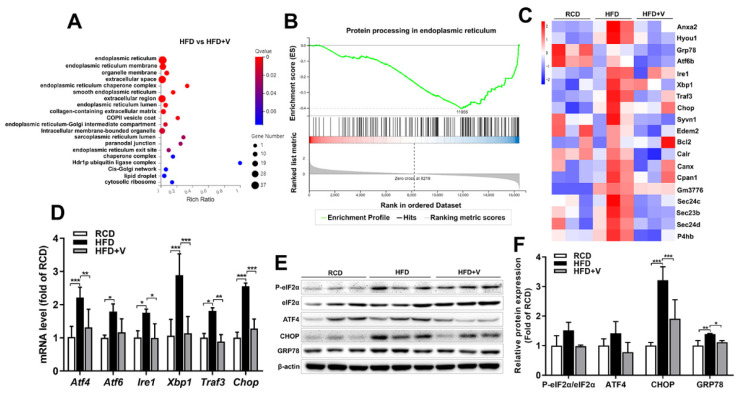
Effect of VOdipic-Cl on the gene expression profile of hepatic ER stress in obese mice. (**A**) GO cellular component enrichment bubble chart showing the functional enrichment pathways of DEGs in the HFD group vs. HFD+V group. (**B**) GSEA-KEGG Gene set enrichment analysis plot showing the DEGs in the HFD group vs. HFD+V group enriched in ER protein synthesis pathway. (**C**) Heat map showing the expression of the genes involved in the ER protein synthesis process. (**D**) The mRNA levels of ER stress-related genes were confirmed by qPCR. (**E, F**) The protein levels of ER stress-related proteins were detected by Western blot. Data are presented as mean ± SD (*n* = 3). * *p* < 0.05, ** *p* < 0.01, *** *p* < 0.001.

To the effect of VOdipic-Cl on the gene expression profile of hepatic ER stress in obese mice, through gene ontology (GO) enrichment analysis, we found that the functions of differential genes between the HFD and HFD+V groups were mainly enriched in ER regulation (Figure 6A). Moreover, GSEA-KEGG pathway analysis showed that VOdipic-Cl significantly inhibited the expression of genes related to ER protein synthesis pathway (Figure 6B). To explore the mechanism of VOdipic-Cl alleviating ER stress, the expression of the DEGs in the ER protein synthesis pathway was visualized in a heatmap. As shown in Figure 6C, VOdipic-Cl down-regulated the expression of genes related to the formation of COPII envelope vesicles (*Anxa2, Sec23b, Sec24c and Sec24d*), and ER stress-related genes, including CCAAT/enhancer binding protein (C/EBP) homologous protein *(Chop)*, glucose-regulated protein 78 *(Grp78)*, activating transcription factor 6 *(Atf6b)*, *Ire1*, *Xbp1*, *Traf3*, *Hyou1*, *Syvn1*, and *Gm3776*. Furthermore, the verification results of the qPCR analysis showed that VOdipic-Cl significantly inhibited the up-regulation of ER stress-related genes induced by HFD (Figure 6D). The analytical results of Western blot showed that VOdipic-Cl also significantly reduced the expression levels of ER stress-related proteins p-eIF2α, ATF4, CHOP and GRP78 (Figure 6E,F).

### 3.7. Effect of VOdipic-Cl on the Gene Expression Profile of Hepatic Inflammation in Obese Mice

**Figure 7:**
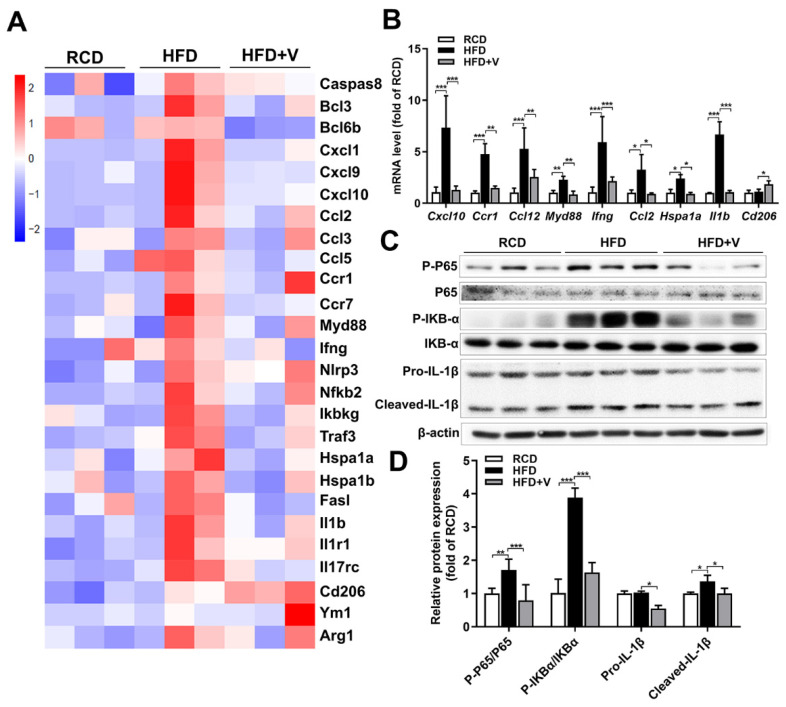
Effect of VOdipic-Cl on the gene expression profile of hepatic inflammation in obese mice. (**A**) Heat map showing the expression of DEGs associated with inflammation. (**B**) The mRNA levels of pro-inflammatory cytokines such as Cxcl10, Myd88, Ccr1, Ccl12, Ifng, Hspa1a, Il1b, Ccl2 and anti-inflammatory cytokine Cd206 were detected by qPCR. (**C**,**D**) The phosphorylation levels of P65, IκB-α, and IL1β expression were detected by Western blot. Data are presented as mean ± SD (*n* = 3). * *p* < 0.05, ** *p* < 0.01, *** *p* < 0.001.

To explore the underlying mechanism for the down-regulated immune pathway in VOdipic-Cl-treated livers in obese mice, the expression profile of some inflammatory response-related genes was investigated. Compared with the RCD group, a heat map of transcriptome analytical results showed that the expression of inflammation-related genes in the HFD group was up-regulated, while it was inhibited by VOdipic-Cl (Figure 7A). The changes in gene expression were further validated by qPCR, which confirmed that HFD induced up-regulation of hepatic CXC motif ligand *(Cxcl10)*, myeloid differentiation factor 88 *(Myd88), Ccr1*, *Ccl12*, *Ifng*, *Hspa1a*, *Il1b* and *Ccl2* gene expression was attenuated by VOdipic-Cl. And the mRNA level of anti-inflammatory cytokine *Cd206* was increased by VOdipic-Cl (Figure 7B). In addition, Western blotting results also showed that VOdipic-Cl inhibited the activation of the NF-κB pathway induced by HFD, decreased the phosphorylation levels of P65 and IκB-α and the expression of IL-1β (Figure 7C,D).

## 4. Discussion

It is well acknowledged that NAFLD is characterized by excessive lipid accumulation and imbalances in lipid metabolism in the liver [2,3]. However, until now, no consensus has been reached on the most effective treatment of NAFLD. It has been demonstrated that vanadium compounds exert insulin-like and antidiabetic effects in vivo and in vitro [30]. Yet, vanadium toxicity is also subject to an ongoing debate, especially the side effects of inorganic vanadium salts [31]. To overcome the negative effects and improve the bioavailability of vanadium, vanadium compounds with dipicolinate (dipic), dipic-NH2, and dipic-Cl as the organic ligand have been synthesized and tested. These compounds have been reported to lower hyperglycemia and hyperlipidemia [17,32]. In the present study, we demonstrated that VOdipic-Cl improves liver lipid metabolism disorders, oxidative stress, ER stress, and inflammation insult, alleviating the development of NAFLD. The protective effects of VOdipic-Cl involved a variety of signaling molecules and pathways.

Dyslipidemia, impaired liver function, and hepatic steatosis are common risk factors for developing NAFLD in obesity and diabetes [33,34]. It has been suggested that the imbalance between fat production and degradation leads to liver steatosis, which is the most direct cause of NAFLD [35]. In the present study, we found that liver injury in the HFD group was accompanied by elevated serum TG, ALT, and AST, and increased liver FFAs levels compared with the RCD group. However, VOdipic-Cl treatment significantly reduced liver weight and the elevation of serum ALT, AST, TG, FFA, TC, and LDL-c, and liver FFAs levels. Liver pathology, including inflammation and adipose infiltration, was improved following VOdipic-Cl treatment, as evidenced by histopathological examination. These findings are consistent with previous reports that bis(maltolato)oxovanadium(IV) (BMOV) and vanadium(III, IV, V)-chlorodipicolinate complexes significantly reduce the levels of plasma or serum TG and TC in streptozotocin (STZ)-induced diabetic rats [36,37]. Therefore, our findings indicate that VOdipic-Cl treatment can mitigate pathological liver steatosis. Furthermore, hepatic oxidative stress is another consequence of NAFLD [35,38]. In the present study, we found that VOdipic-Cl significantly reduced hepatic 3′-NT and MDA levels. This reduction was associated with increased GSH/GSSG, suggesting that VOdipic-Cl attenuates HFD-induced lipid peroxidation and oxidative stress in the livers of the NAFLD model. However, more evidence and molecular mechanism investigations are still needed considering the multipathological factors in the progression of fatty liver.

To further investigate the molecular mechanism of the benefits of VOdipic-Cl on NAFLD, we explored the mRNA expression profile of hepatic tissue. Transcriptomic analysis is a precise and sensitive tool for measuring global gene expression profile expression [39]. In this study, a transcriptome profiling analysis was performed to identify genes that are differentially expressed in HFD vs. HFD+V group. Most DEGs were enriched in metabolism pathways, genetic information processing, and immune pathways, such as steroid biosynthesis, fatty acid metabolism, PPAR signaling pathway, and protein processing in the endoplasmic reticulum. A previous study has demonstrated that the expression of genes dysregulated in diabetes is normalized by vanadyl sulfate treatment, which belongs to lipid metabolism, oxidative stress, protein breakdown and biosynthesis, complement system, and signal transduction [40]. Thus, our findings indicate that VOdipic-Cl remarkably inhibits and regulates the mRNA expression levels of hepatic lipogenesis genes and liver enzymes related to lipid metabolism in the livers of hyperlipidemia mice.

It is well documented that the sources of liver FFAs mainly include non-esterified fatty acids (NEFA) secreted by adipose tissue, de novo lipogenesis (DNL), and dietary intake [35,41]. Of the FFAs found in the liver of NAFLD patients, 59% are derived from plasma FFAs, whereas 15% and 26% are derived from dietary fat and de novo lipogenesis, respectively [41]. Several studies provide evidence that SREBP-1c, C/EBPα, and PPARγ regulate the de novo lipogenesis and promote fatty acid uptake by activating the transcription of genes related to fatty acid transport and synthesis, such as fatty acid translocase *(Fat/Cd36)*, acetyl-coa carboxylase *(Acc),* and *Fas* [42,43,44]. In the present study, VOdipic-Cl significantly down-regulated genes related to lipid synthesis, including *Acss3*, *Elovl5*, *Gpat3*, *Dgat1*, *Fas*, *Srebp1c*, *Pparγ*, *C/EBPα,* and *Insig2* in the livers of obese mice. Moreover, the mRNA expression levels of genes related to fatty acid transport and lipolysis, including *Apoa1, Fabp4, Fabp5, Atgl, Hsl,* and *Ucp2* were also remarkably up-regulated. In agreement with previous studies [45,46], our study also showed that VOdipic-Cl improves liver lipid metabolism disorders by down-regulating the expression of PPARγ, C/EBPa, SREBP-1c and FAS proteins related to lipid synthesis. It has been reported that esterification of FFA to TG and β-oxidation of FFAs are two main metabolic pathways of FFAs in the liver [47]. FAAs are converted into TGs and stored in lipid droplets, some of which are exported into the blood as very-low-density lipoprotein, causing hypertriglyceridemia [48,49]. In mammals, perilipins (PLIN) are representative structural proteins of lipid droplets, mainly PLIN1 and PLIN2, which sequester lipids by protecting lipid droplets from lipase action [50,51]. Especially, PLIN2 is the most abundant PLIN family protein in the liver, which regulates the interaction between ATGL and lipid droplets [52]. When ATGL or HSL is lost, the accumulation of TG and lipid toxicity in the HFD-induced livers of mice are aggravated [53,54]. After TG is catabolized into glycerol and fatty acids, ultra-long-chain fatty acids are transported to mitochondria by CPT1 and converted to acylcarnitine [55,56]. In this study, we observed that VOdipic-Cl promotes TG catabolism by down-regulating the expression of *Plin2* and up-regulating the expression of *Atgl* and *Hsl* in the livers of obese mice. Moreover, VOdipic-Cl promoted the β-oxidation of fatty acids by up-regulating the expression of *Cpt1* and *Ucp2* in mouse liver. Thus, our results validated that VOdipic-Cl displays a dual modulatory role in inhibiting lipogenesis and the induction of mitochondrial β oxidation of FFAs.

On the other hand, dysregulation of lipid homeostasis in hepatocytes leads to transient generation or accumulation of toxic lipids that result in ER stress with inflammation, hepatocellular damage, and apoptosis [57,58]. ER stress activates the unfolded protein response (UPR), classically viewed as an adaptive pathway to maintain protein folding homeostasis [57,58]. Accumulation of unfolded protein in the ER increases ROS production and evokes ER stress, which involves the development of NAFLD [6]. Definitely, the UPR is activated via inositol-requiring enzyme 1 (IRE1), PKR-like ER kinase (PERK) and activating transcription factor 6 (ATF6) in the regulation of hepatic steatosis and the cellular response to lipotoxic stress [9,59]. As indicators of ER stress, GPR78 and CHOP are included in lipid metabolism and tissue injury in NAFLD [60]. In this NAFLD model, the increased expression of ER stress-related genes, including *Chop*, *Grp78*, *Atf6b*, *Ire1*, *Xbp1*, *Traf3*, *Hyou1*, *Syvn1*, and *Gm3776*, indicates the induction of ER stress in NAFLD. However, these elevated expressions of genes in mouse livers were down-regulated after VOdipic-Cl treatment. Bis(ethylmaltolato)oxidovanadium (IV) (BEOV) ameliorates ER stress and neuronal apoptosis by regulating PPARγ in mouse Alzheimer’s disease models [61]. BMOV protects diabetic cardiomyopathy by counteracting ROS and ER stress in the hearts of STZ-induced diabetic rats [20]. In light of these similarities, VOdipic-Cl treatment may have attenuated hepatic ER stress in NAFLD by inhibiting the expression of UPRs.

It has been demonstrated that the hepatocellular damage characterized by inflammation is linked to ER dysfunction, resulting from toxic lipids termed as lipotoxic ER stress [62,63]. A recent study has demonstrated that lipotoxic sublethal ER stress response leads to the release of pro-inflammatory extracellular vesicles by lipotoxic hepatocytes [64]. In the development of NAFLD, ER stress aggravated hepatic inflammation by activating the NF-κB pathway, increasing the expression of pro-inflammatory cytokines [65]. In the present study, our transcriptome data showed that VOdipic-Cl significantly down-regulated the expression of genes related to alternative pathways of inflammation (*Cxcl10*, *Ccr1*, *Ccl12*, *Ifng*, *Il1b* and *Ccl2*) in the livers of obese mice. Also, the inflammatory/immune pathways were deeply influenced by VOdipic-Cl, including protein processing in the endoplasmic reticulum pathway, steroid biosynthesis pathway, and retinol metabolism pathway. Additionally, VOdipic-Cl inhibited the activation of the hepatic NF-κB pathway, as evidenced by decreasing phosphorylation of P65 and IκB-α and expression of IL-1β. ER stress has been found to activate the NF-κB pathway and promote liver inflammation [9]. IRE1α activates IκB and JNK, promotes the entry of NF-κB into the nucleus, up-regulates the expression of pro-inflammatory cytokines such as TNFα, IL-6 and IL-1β, and promotes inflammation [66,67]. PERK also regulates NF-κB and apoptosis through the activation of eIF2a-ATF4-CHOP ax-is of UPR. Activation of PERK downregulates global protein translation, which preferentially affects IκB expression over NF-κB as IκB, has a shorter half-life, enabling NF-κB to translocate. ATF6 also positively affects NF-κB activation via mTOR/AKT signaling [68]. Our finding suggested that the anti-inflammatory effect of VOdipic-Cl is associated with its inhibition of ER stress.

## 5. Conclusions

In summary, our present work demonstrates that VOdipic-Cl treatment not only improves hyperlipidemia and oxidative stress, but also reduces ER stress and inflammatory response under the HF diet style. These beneficial effects could be attributed to regulating lipid metabolism disorders in the liver. In addition, VOdipic-Cl effectively enriches the gene sets involved in lipid metabolism, ER stress, and immune response, providing new insights into the molecular mechanism of alleviating the effect of VOdipic-Cl against hepatic steatosis.

## Figures and Tables

**Table 1:** The top 10 up- and down-regulated genes induced by HFD.

Gene ID	Gene Name	log2(HFD/RCD)	Q-value
**Top 10 up-regulated genes**			
12683	Cidea	9.023595	8.99 × 10^−12^
228677	Sptlc3	7.503749	3.56 × 10^−5^
13094	Cyp2b9	6.854523	3.98 × 10^−8^
13086	Cyp2a4	6.713696	6.43 × 10^−6^
268958	Capn11	5.638241	0.013663
93695	Gpnmb	5.318517	3.74 × 10^−4^
20753	Sprr1a	5.211094	0.019481
239463	Fam83a	5.151803	1.80 × 10^−9^
11537	Cfd	5.049939	0.002032
68393	Mogat1	5.009104	3.35 × 10^−20^
**Top 10 down-regulated genes**			
59012	Moxd1	−9.08269	3.80 × 10^−13^
13099	Cyp2c40	−6.86642	1.10 × 10^−32^
100043108	Cyp2c69	−6.54387	3.12 × 10^−14^
14282	Fosb	−4.1573	6.04 × 10^−5^
20500	Slc13a2	−4.13595	8.11 × 10^−8^
20704	Serpina1e	−3.59604	2.78 × 10^−80^
100559	Ugt2b38	−3.54827	5.53 × 10^−47^
77596	Adgrf1	−3.46736	1.10 × 10^−32^
13082	Cyp26a1	−3.3903	3.96 × 10^−12^
233799	Acsm2	−3.22653	0.005442

**Table 2:** The top 10 up- and down-regulated genes induced by VOdipic-Cl.

Gene ID	Gene Name	log2(HFD+V/HFD)	Q-value
**Top 10 up-regulated genes**			
13035	Ctsg	6.260156209	0.027929352
27028	Ermap	4.653195976	0.042060535
50701	Elane	4.272854575	0.014677
59012	Moxd1	4.251877958	1.53 × 10^−4^
55985	Cxcl13	3.583918049	0.025926151
18022	Nfe2	3.420277595	0.002922876
12700	Cish	3.085936054	1.43 × 10^−30^
13099	Cyp2c40	3.029913721	4.20 × 10^−4^
57349	Ppbp	2.980473843	0.036379545
105246824	Gm42048	2.352530182	4.30 × 10^−5^
**Top 10 down-regulated genes**			
20753	Sprr1a	−6.41864	0.008521
14857	Gsta1	−4.58046	0.021441
12164	Bmp8b	−4.4467	0.04983
13844	Ephb2	−4.43179	0.023676
100042295	Gm3776	−4.03746	0.030474
13086	Cyp2a4	−3.07994	0.005285
53321	Cntnap1	−2.42271	1.48 × 10^−5^
239463	Fam83a	−2.41749	0.028261
76293	Mfap4	−2.09718	0.013435
12053	Bcl6	−2.07082	2.32 × 10^−12^

**Table 3:** Significantly enriched KEGG pathway of DEGs in RCD vs. HFD group. * Rich ratio is defined as the amount of differentially expressed genes enriched in the pathway/amount of all genes in the background gene set.

Pathway ID	Pathway Name	Gene Number	Rich Ratio *	Q value
ko3010	Ribosome	42	0.304347826	1.62 × 10^−22^
ko4610	Complement and coagulation cascades	24	0.258064516	8.68 × 10^−11^
ko830	Retinol metabolism	23	0.252747253	2.87 × 10^−10^
ko140	Steroid hormone biosynthesis	20	0.227272727	3.70 × 10^−8^
ko3320	PPAR signaling pathway	20	0.229885057	3.70 × 10^−8^
ko5204	Chemical carcinogenesis	20	0.210526316	1.30 × 10^−7^
ko1212	Fatty acid metabolism	15	0.245901639	1.24 × 10^−6^
ko71	Fatty acid degradation	10	0.2	0.001626099
ko983	Drug metabolism—other enzymes	13	0.147727273	0.003072717
ko980	Metabolism of xenobiotics by cytochrome P450	11	0.164179104	0.003637073
ko20	Citrate cycle (TCA cycle)	6	0.1875	0.03105017
ko4141	Protein processing in endoplasmic reticulum	15	0.091463415	0.06166278

**Table 4:** Significantly enriched KEGG pathway of DEGs in HFD vs. HFD+V group. * Rich ratio is defined as the amount of differentially expressed genes enriched in the pathway/amount of all genes in the background gene set.

Pathway ID	Pathway Name	Gene Number	Rich Ratio *	Q value
ko100	Steroid biosynthesis	5	0.25	1.06 × 10^−4^
ko4141	Protein processing in endoplasmic reticulum	10	0.06097561	1.40 × 10^−4^
ko830	Retinol metabolism	5	0.054945055	0.050931566
ko4950	Maturity onset diabetes of the young	3	0.111111111	0.050931566
ko5204	Chemical carcinogenesis	5	0.052631579	0.050931566

## Data Availability

The data presented in this study are available on reasonable request from the corresponding author. The sequencing data for clean reads generated by this study have been deposited in the NCBI Sequence Read Archive (SRA) database (accession number: PRJNA540011).

## Supplementary Materials

The following supporting information can be downloaded at: https://www.mdpi.com/article/10.3390/antiox11061093/s1, Figure S1: Analysis of differentially expressed genes between RCD group and HFD group; Figure S2: Analysis of KEGG pathways. Table S1: The quantitative real-time PCR primer information; Table S2: Detail information for antibodies.
