# Supplementary material for: Vanadium(IV)-Chlorodipicolinate Protects against Hepatic Steatosis by Ameliorating Lipid Peroxidation, Endoplasmic Reticulum Stress, and Inflammation
Source: Antioxidants (Basel). 2022 May 31;11(6):1093. doi: 10.3390/antiox11061093 (PMC9220021; doi:10.3390/antiox11061093)
Supplement: Supplementary file 1 [file antioxidants-11-01093-s001.zip › antioxidants-1751906-supplementary.pdf]

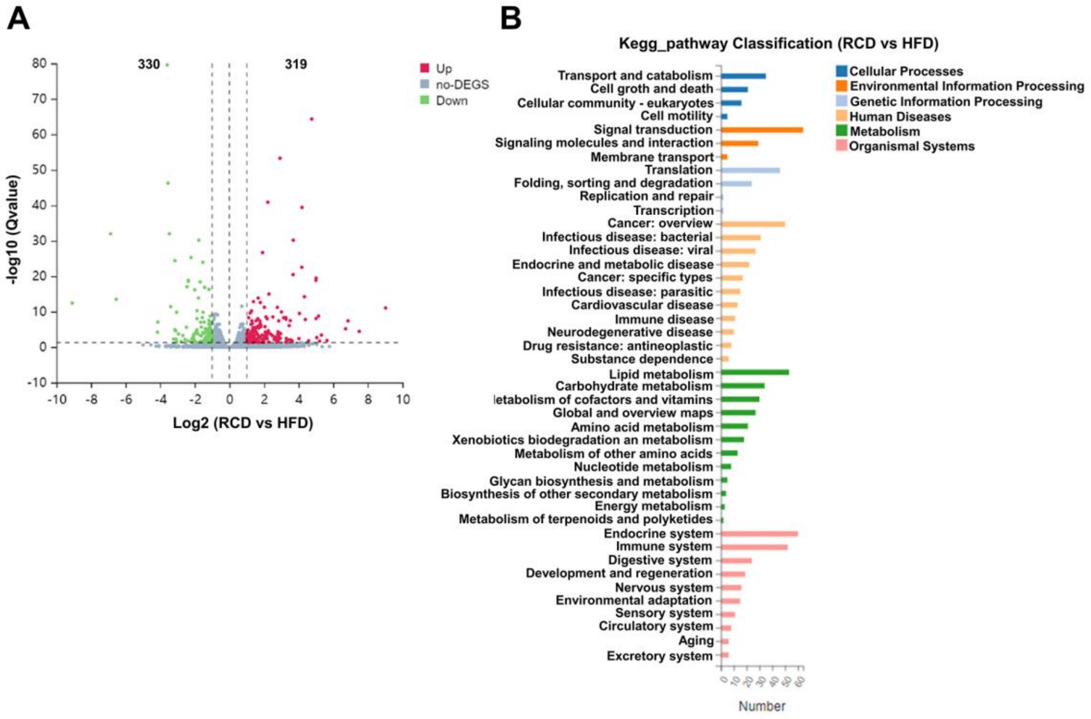

Figure S1: Analysis of differentially expressed genes between RCD group and HFD group. (A) Volcano plot shows the fold change of DEGs of RCD vs HFD group. (B) KEGG pathway classification of DEGs from RCD vs HFD group.

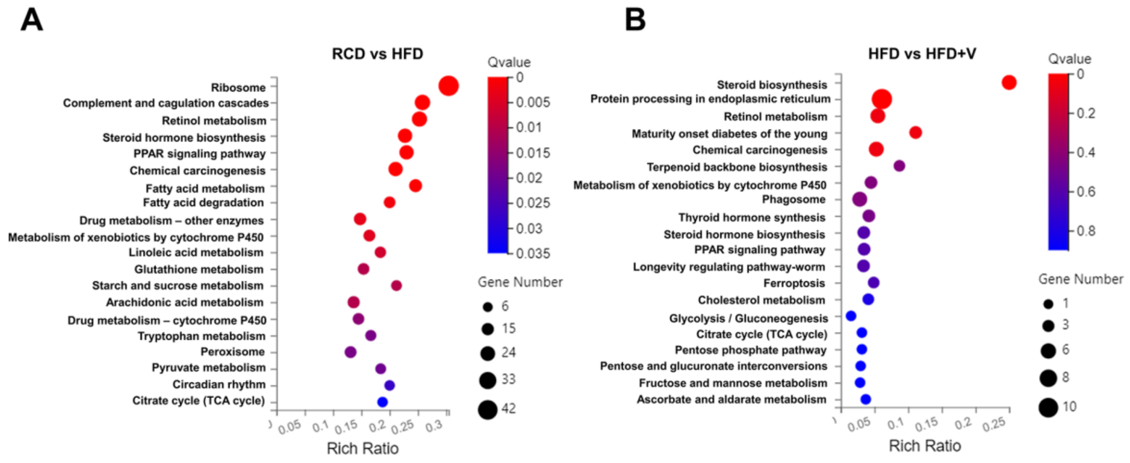

Figure S2. Analysis of KEGG pathways. The advanced bubble chart shows the top 20 enriched KEGG pathways of the DEGs in the RCD vs HFD group (A), and HFD vs HFD+V group (B).

Table S1. The quantitative real-time PCR primer information.

Gene name	Forward primer (5′-3′)	Reverse primer (5′-3′)
Ppary	CGCTGATGCACTGCCTATGA	AGAGGTCCACAGAGCTGATTCC
Srebp-1c	GGCAGGCTTTGAAGACGAA	CACGACAAGGACTGAGGGAC

Cebp/a	AACGCAACAACATCGCGGT	GCGGTCATTGTCACTGGTCAA
Fas	AGTTTCATGAACCCGCCTC	GCAGACATGCTGTGGATCTG
Ppara	GTGGGTGGTTGAATCGTGAG	GCAGTGGAGTTTGGGTTGG
Atgl	TGGATGGCGGCATTTCAGACA	TGACGCGAAGCTCGTGGATGTT
Hsl	GGAGAGAGTCTGCAGGAACG	CCTGCAAGAGTATGTCACGC
Cpt1	AGTGGCCTCACAGACTCCAG	GCCCATGTTGTACAGCTTCC
Ucp2	TCCTGCTACCTCCCAGAAGA	TGAGACCTCAAAGCAGCCTC
Atf4	ATGGCGCTCTTCACGAAATC	ACTGGTCGAAGGGGTCATCAA
Atf6	GACTCACCCATCCGAGTTGTG	CTCCCAGTCTTCATCTGGTCC
Xbp1	AGCAGCAAGTGGTGGATTTG	GAGTTTTCTCCCGTAAAAGCTGA
Ire1	ACACCGACCACCGTATCTCA	CTCAGGATAATGGTAGCCATGTC
Traf3	CAGCCTAACCCACCCCTAAAG	TCTTCCACCGTCTTCACAAAC
Chop	CTGGAAGCCTGGTATGAGGAT	CAGGGTCAAGAGTAGTGAAGGT
Grp78	ACTTGGGGACCACCTATTCCT	ATCGCCAATCAGACGCTCC
Cxcl10	CCAAGTGCTGCCGTCATTTTC	GGCTCGCAGGGATGATTTCAA
Ccr1	CTCATGCAGCATAGGAGGCTT	ACATGGCATCACCAAAAATCCA
Ccl12	ATTTCCACACTTCTATGCCTCCT	ATCCAGTATGGTCCTGAAGATCA
Ifng	ATGAACGCTACACACTGCATC	CCATCCTTTTGCCAGTTCCTC
Myd88	AGGACAAACGCCGGAACTTTT	GCCGATAGTCTGTCTGTTCTAGT
Hspa1a	TGGTGCAGTCCGACATGAAG	GCTGAGAGTCGTTGAAGTAGGC
Il1β	GCAACTGTTCCTGAACTCAACT	ATCTTTTGGGGTCCGTCAACT
Ccl2	TCTTCCTGCTCACATTACCAT	CACAGCATGAACAATAGCCAA
Cd206	TTGGACGGATAGATGGAGGG	CCAGGCAGTTGAGGAGGTTC
actin	GGCTGTATTCCCCTCCATCG	CCAGTTGGTAACAATGCCATGT

Table S2. Detail information for antibodies**.**

Targeted protein	Cat. no.	Source	Provider
FAS	3189	rabbit	Cell Signaling Technology
PPARγ	2435S	rabbit	Cell Signaling Technology
ATF4	11815S	rabbit	Cell Signaling Technology
eIF2a	5324	rabbit	Cell Signaling Technology
p-eIF2a	9721S	rabbit	Cell Signaling Technology
IL-1β	12242S	rabbit	Cell Signaling Technology
P-P65	3033S	rabbit	Cell Signaling Technology
ATGL	sc-365278	mouse	Santa Cruz Biotechnology
CPT1	sc-393070	mouse	Santa Cruz Biotechnology
CHOP	sc-7351	mouse	Santa Cruz Biotechnology
GRP78	sc-166490	mouse	Santa Cruz Biotechnology
IKB-a	sc-371	Rabbit	Santa Cruz Biotechnology
P-IKB-a	sc-8404	mouse	Santa Cruz Biotechnology
CEBP/a	ab40764	rabbit	Abcam PLC
SREBP-1c	#P02A751L	mouse	Jinpulai Biotechnology Co., Ltd.
HSL	ab45422	rabbit	Abcam PLC
P65	32705	rabbit	Signalway antibody
